# Risk stratification based on J-ACCESS risk models with myocardial perfusion imaging: Risk versus outcomes of patients with chronic kidney disease

**DOI:** 10.1007/s12350-018-1330-8

**Published:** 2018-06-12

**Authors:** Kenichi Nakajima, Satoko Nakamura, Hiroki Hase, Yasuchika Takeishi, Shigeyuki Nishimura, Yuhei Kawano, Tsunehiko Nishimura

**Affiliations:** 1 Department of Nuclear Medicine, Kanazawa University Hospital, Kanazawa, 920-8641 Japan; 2 Division of Hypertension and Nephrology, National Cerebral and Cardiovascular Center, Suita, Japan; 3 Kansai University of Welfare Sciences, Kashihara, Japan; 4 Department of Nephrology, Toho University Ohashi Medical Center, Tokyo, Japan; 5 Department of Cardiovascular Medicine, Fukushima Medical University, Fukushima, Japan; 6 Saitama Medical University International Medical Center, Hidaka, Japan; 7 Department of Medical Technology, Teikyo University, Fukuoka, Japan; 8 Graduate School of Medical Science, Kyoto Prefectural University of Medicine, 465 Kajiicho, Kawara-machi Hirokoji, Kamigyo-ku, Kyoto, 602-8566 Japan

**Keywords:** Prognosis, cardiac events, multivariable logistic analysis, heart failure, C-reactive protein

## Abstract

**Background:**

This study aimed to validate the accuracy of major-event risk models created in the multicenter J-ACCESS prognostic study in a new cohort of patients with chronic kidney disease (CKD).

**Methods and Results:**

Three multivariable J-ACCESS risk models were created to predict major cardiac events (cardiac death, non-fatal acute coronary syndrome, and severe heart failure requiring hospitalization): Model 1, four variables of age, summed stress score, left ventricular ejection fraction and diabetes; Model 2 with five variables including estimated glomerular filtration rate (eGFR, continuous); and Model 3 with categorical eGFR. The validation data used three-year (3y) cohort of patients with CKD (*n* = 526, major events 11.2%). Survival analysis of low (< 3%/3y), intermediate (3% to 9%/3y), and high (> 9%/3y)-risk groups showed good stratification by all three models (actual event rates: 3.1%, 9.9%, and 15.9% in the three groups with eGFR ≥ 15 mL/min/1.73 m^2^, *P* = .0087 (Model 2). However, actual event rates were equally high across all risk groups of patients with eGFR < 15 mL/min/1.73 m^2^.

**Conclusion:**

The J-ACCESS risk models can stratify patients with CKD and eGFR ≥ 15 mL/min/1.73 m^2^, but patients with eGFR < 15 mL/min/1.73 m^2^ are potentially at high risk regardless of estimated risk values.

**Electronic supplementary material:**

The online version of this article (10.1007/s12350-018-1330-8) contains supplementary material, which is available to authorized users.

## Introduction

Myocardial perfusion abnormalities and left ventricular (LV) dysfunction can predict major cardiac events, and single-photon emission-computed tomography (SPECT) myocardial perfusion imaging (MPI) can provide vital information for prognosis.^1^^–^4 Large-scale single- and multi-center studies have found that a larger defect size on MPI, LV dysfunction such as lower LV ejection fraction (LVEF) and higher LV volumes are generally associated with an increased incidence of cardiovascular events. Therefore, these factors have been included as statistically significant variables in uni- and multi-variable analyses even in Japanese multi-center cohort studies.^5^^–^7 In addition, since associated conditions such as diabetes and chronic kidney disease (CKD) are also major predictors of cardiovascular events, the estimation of clinical risk factors could play an important role in clinical practice.^7^,^8^

Risk models for estimating cardiac events have been developed using score-related event risks^9^ and calculations of event risk (%).^10^,^11^ However, although multifactorial effects on cardiovascular events have been demonstrated, few studies have evaluated the accuracy of these multivariable risk models to predict such events in Japanese populations by comparing predicted and actual outcomes.

J-ACCESS prognostic studies have been ongoing in Japan since 2001, and event risk models to estimate major cardiac events, including cardiac death, non-fatal myocardial infarction (MI) and severe heart failure (HF) requiring hospitalization, have been created for clinical applications.^10^ These J-ACCESS risk models incorporated the risk factors of age, LVEF, and perfusion defects under stress determined using summed stress score (SSS), diabetes, and subsequently estimated glomerular filtration rates (eGFR).^11^ The J-ACCESS-3 study was an independent multi-center study in Japan in patients with CKD, and three-year outcomes were evaluated in patients with eGFR < 50 mL/min/1.73 m^2^.^12^,^13^

The hypothesis of the present study is that risk estimated using the model from the J-ACCESS study could be in agreement with the actual outcome of the J-ACCESS-3 study, which was used as a validation population. The present study also aimed to determine the effect of eGFR on associations between risk models and outcomes.

## Methods

### Risk Models Created in J-ACCESS Study

Table 1 summarizes the demographics of the derivation population in the J-ACCESS study of the risk model. The study group consisted of 4,031 patients who were assessed by ^99m^Tc-tetrofosmin SPECT MPI. The inclusion criteria were ≥ 20 years of age and undergoing stress and rest MPI due to suspected or known ischemic heart diseases. Patients with the onset of MI or unstable angina pectoris within 3 months, cardiomyopathy, and heart failure with class III or higher New York Heart Association classification were excluded. Perfusion defects were semi-quantified in a 17-segment model using a scale from 0 (normal) to 4 (absent) for each segment and then summed stress/rest/difference scores (SSS/SRS/SDS) were calculated.

**Table 1:** Demographics of derivation and validation populations for risk models

	Derivation population	Derivation population	Validation population
	Model 1	Models 2 and 3	J-ACCESS-3 (CKD)
Number	4,031	2,395	526
Age (years)	65.9 ± 10.1	66.1 ± 10.0	71.6 ± 10.9
Male gender (%)	64%	64%	67%
Typical chest pain (%)	46%	46%	34%
Body mass index (kg/m^2^)	23.7 ± 3.2	23.7 ± 3.3	24.1 ± 4.1
History of myocardial infarction (%)	29%	31%	0%
History of revascularization (%)	36%	37%	0%
Diabetes mellitus (%)	29%	33%	42%
Hypertension (%)	55%	56%	91%
Dyslipidemia (%)	47%	51%	49%
Current smoking (%)	16%	16%	6%
eGFR (mL/min/1.73 m^2^)	Not used	67.3 ± 30.7	29.0 ± 12.8
Summed stress score	8.7 ± 11.2	9.0 ± 11.5	1.9 ± 3.8
Summed rest score	7.3 ± 10.6	7.6 ± 11.0	1.1 ± 3.0
Summed difference score	1.4 ± 3.8	1.5 ± 3.8	0.8 ± 1.8
Left ventricular ejection fraction at rest (%)	62 ± 14	61 ± 14	62 ± 15
End-diastolic volume at rest (mL)	85 ± 36	85 ± 38	91 ± 39
End-systolic volume at rest (mL)	36 ± 29	37 ± 31	39 ± 31
Reference No.	10	11	13

*CKD*, chronic kidney disease; *eGFR*, estimated glomerular filtration rate

During 3 years of follow-up, a total of 175 (4.3%) of the patients developed the major events of cardiac death (*n* = 54; 1.3%), non-fatal MI (*n* = 37; 0.9%) and severe HF requiring hospitalization (*n* = 93; 2.3%).

Three-year risk of events (p, %/3 y) was calculated using the multivariable logistic model:

$$ {\text{p }} = { 1}00 \, / \, \left( { 1 { } + {\text{ Exp}}\,\left[ { - {\text{b}}(0)\, + \,\sum {\left( {{\text{b}}\left( {\text{i}} \right) \, \times {\text{ x}}\left( {\text{i}} \right)} \right)} } \right]} \right)\, ( {\text{\% )}}, $$

where b(i) is a parameter estimate of a predictive variable x(i). To create the logistic risk models, patients who were censored alive within the 3 year follow-up were not included.

#### Model 1: four-parameter model without eGFR

Multivariable logistic analysis of the 4,031 individuals showed that SSS (with four categorical variables of 0-3, 4-8, 9-12, and ≥ 13 by a 20-segment model; revised later to 0-3, 4-7, 8-11, and ≥ 12 by a 17-segment model), LVEF, age, and diabetes were significant variables (Table 2).^10^

**Table 2:** Parameter estimates based on multivariable logistic analysis in three risk models

	Model 1		Model 2		Model 3	
	Coefficients	Comments	Coefficients	Comments	Coefficients	Comments
Age	0.0558	Continuous	0.0582	Continuous	0.0572	Continuous
Diabetes mellitus	0.8858	Yes (1)/no (0)	0.7998	Yes (1)/no (0)	0.7742	Yes (1)/no (0)
LVEF	− 0.0475	Continuous	− 0.0359	Continuous	− 0.0363	Continuous
SSS	0.1941	Categorical (0–3)	0.697	Categorical (0, 1)	0.2305	Categorical (0–3)
eGFR	–	None	− 0.0151	Continuous	− 0.3522	Categorical (1–5)
Intercept	− 4.8125		− 4.699		− 4.5815	
Number of patients for creating models	4,031		2,453		2,453	

*eGFR*, estimated glomerular filtration rate; *LVEF*, left ventricular ejection fraction; *SSS*, summed stress score

#### Model 2: five-parameter model with continuous eGFR

Subsequent analysis based on patients with information about eGFR (*n* = 2,395) clarified the importance of including CKD or eGFR. Therefore, all coefficients were determined based on similar logistic analysis with five variables (Table 2).^11^ SSS was classified as 0 (< 8) and 1 (≥ 8).

#### Model 3: five-parameter model with categorical eGFR and four SSS classes

Model 3 was used in Heart Risk View software [Nihon Medi-Physics, Tokyo, Japan], which was a revision of Model 2 that included categorical variables and was intended for practical application to clinical situations. The model included eGFR (mL/min/1.73 m^2^) classified as 1 (< 30), 2 (≥ 30, < 45), 3 (≥ 45, < 60), 4 (≥ 60, < 90), and 5 (≥ 90); and SSS was classified as 0 (≤ 3), 1 (4-7), 2 (8-11), and 3 (≥ 12).

### Patients in the Validation Population

The principal design and results of J-ACCESS-3 are described elsewhere.^14^ A total of 549 patients were registered from 62 institutions between 2009 and 2010. The inclusion criteria included patients aged ≥ 20 years who were scheduled to undergo stress-rest electrocardiographic gated MPI due to having suspected ischemic coronary artery disease (CAD), eGFR < 50 mL/min/1.73 m^2^, and one or more of the seven risk factors for CAD (hypertension, diabetes, dyslipidemia, peripheral vascular diseases, currently smoking, family history of juvenile CAD, and history of ischemic stroke). Chronic kidney disease was defined based on the Japanese equation for eGFR (mL/min/1.73 m^2^).^15^ The exclusion criteria included hemodialysis or peritoneal dialysis, severe valvular heart disease requiring surgical treatment, cardiomyopathies, prior diagnosis of angina pectoris or MI, and a history of revascularization, percutaneous coronary intervention (PCI), or a coronary artery bypass graft surgery. A total of 526 patients who had complete sets of variables for the risk model were analyzed.

### MPI Protocol

Either 1-day (96%) or 2-day (4%) protocols were applied in pharmacological stress MPI studies using a standard protocol of ^99m^Tc-tetrofosmin and Anger-type cameras. Pharmacological stress studies proceeded using adenosine (91%), dipyridamole, or adenosine triphosphate. The average administered doses of ^99m^Tc-tetrofosmin for the initial and second studies were 312 and 689 MBq, respectively. Chest pain (*n* = 7, 1.3%), ST depression (*n* = 3, 0.6%), atrioventricular block (the second degree or worse) (*n* = 2, 0.4%), and serious arrhythmia (*n* = 2, 0.4%) developed during the pharmacological stress study. Both LVEF and LV volumes were quantified using QGS software (Cedars-Sinai Medical Center, Los Angeles, CA, USA).

### Evaluation of MPI Findings

An interpretation committee comprising seven nuclear cardiology experts objectively analyzed all data from standard SPECT slices including SSS, SRS, and SDS. Thresholds for defect scoring were based on a normal database derived from the Japanese Society of Nuclear Medicine working group database.^16^

### Events During Follow-Up

The endpoints of the J-ACCESS-3 study were major cardiac events comprising cardiac death, sudden cardiac death, non-fatal MI, and hospitalization to treat HF. Sudden death was defined as death of unknown cause at 24 hours after occurrence. These major cardiac events were confirmed at each institution based mainly on medical charts, along with written questionnaires and telephone interviews. During the 3-year follow-up, 4.8% and 3.6% of the patients were lost to the J-ACCESS and J-ACCESS 3 studies, respectively.

### Ethical Approval

The Institutional Review Boards of all participating hospitals approved both the J-ACCESS and J-ACCESS-3 studies, both of which also complied with the Ethical Guidelines for Epidemiological Research in Japan.^14^ All patients provided written informed consent to participate in the study.

### Statistical Analysis

Data are expressed as mean ± standard deviation (SD) or median and ranges for non-normal distribution. Continuous variables were compared using *T* tests and analyses of variance. Categorical data between groups were compared using χ^2^ tests. Since C-reactive protein (CRP) was one of the major independent prognostic factors at a threshold of 0.3 mg/mL,^13^ the frequency of high CRP was also examined in each group. Calculated differences in cardiac event risks among groups within the three models were compared using Kaplan–Meier survival analysis. Appropriate thresholds for three models were determined from the three risk groups of patients by analyzing receiver-operating characteristics (ROC) curves and histograms; < 3%/3 years (1%/year) for low-risk patients and > 9% for high-risk patients (corresponding to the lower and upper quartiles of the event risks). All data were statistically analyzed using JMP software version 12.2 (SAS Institute Inc., Cary, NC, USA). A *P* value of < 0.05 was considered to indicate a significant difference.

## Results

### Demographics of Patients

Table 1 summarizes the background conditions for the model-derivation groups and the J-ACCESS-3 study. The validation population did not include any patients with a history of either MI or revascularization. The eGFR values in Model 2 and J-ACCESS-3 were 67.3 ± 30.7 and 29.0 ± 12.8 mL/min/1.73 m^2^ (*P* < .0001), respectively. The SSS, SRS, and SDS were lower in J-ACCESS-3 than in the Model 2 derivation populations (*P* < .0001 for all).

### Major Cardiovascular Events

Out of 526 patients 59 (11.2%) patients developed cardiac events, and 13 (2.5%) had cardiac or sudden death. The events in these 59 patients comprised HF treated upon admission (*n* = 42) sudden death (*n* = 6), cardiac death (*n* = 3), death due to HF upon admission (*n* = 3), non-fatal MI (*n* = 2), non-fatal MI and HF upon admission (*n* = 2), and non-fatal MI and subsequent cardiac death (*n* = 1). We found lower values for LVEF (52.1% ± 14.8% vs 62.7% ± 14.7%, *P* < .0001) and eGFR (25.0 ± 12.4 vs 29.4 ± 12.7 mL/min/1.73 m^2^, *P* = .026), and higher values for EDV (113.9 ± 43.6 vs 88.1 ± 38.3 mL, *P* = .0004), ESV (58.1 ± 35.3 vs 37.0 ± 30.2 mL, *P* = .0003), SSS (3.7 ± 6.7 vs 1.6 ± 3.2, *P* = .046), and SRS (2.8 ± 6.0 vs 0.9 ± 2.4, *P* = .040), in a group that developed HF as the first event (*n* = 45) compared with a group that did not develop events (*n* = 466). The SDS did not significantly differ between these groups (1.0 ± 2.1 vs 0.8 ± 1.8, *P* = ns).

Renal events of hemodialysis and peritoneal dialysis developed in 45 (8.6%) and 4 (0.8%) patients, respectively. Patients with eGFR < 15 mL/min/1.73 m^2^ (*n* = 99) showed more frequent renal events including 40 (40.4%) with hemodialysis and 4 (4.0%) with peritoneal dialysis.

### Distribution of Risk Values Calculated by Three Models

The median risk values were 3.3% (range 0.2%-36.6%), 5.3% (0.25%-44.2%), and 5.0% (0.25%-44.5%) for Models 1, 2, and 3, respectively, and were highest for Model 2 (Models 1 vs 2, and 1 vs 3, both *P* < .0001; Model 2 vs 3 *P* = .0041). High correlation was observed for risk values between Models 1, 2, and 3 showing Risk by Model 2 = 1.5 + 1.21 × Risk by Model 1 (*R*^2^ = 0.92, *P* < .0001) and Risk by Model 2 = 0.40 + 1.03 × Risk by Model 3 (*R*^2^ = 0.97, *P* < .0001).

### Estimation of Cardiac Event Risk by Models 1, 2, and 3 with Respect to eGFR

**Figure 1:**
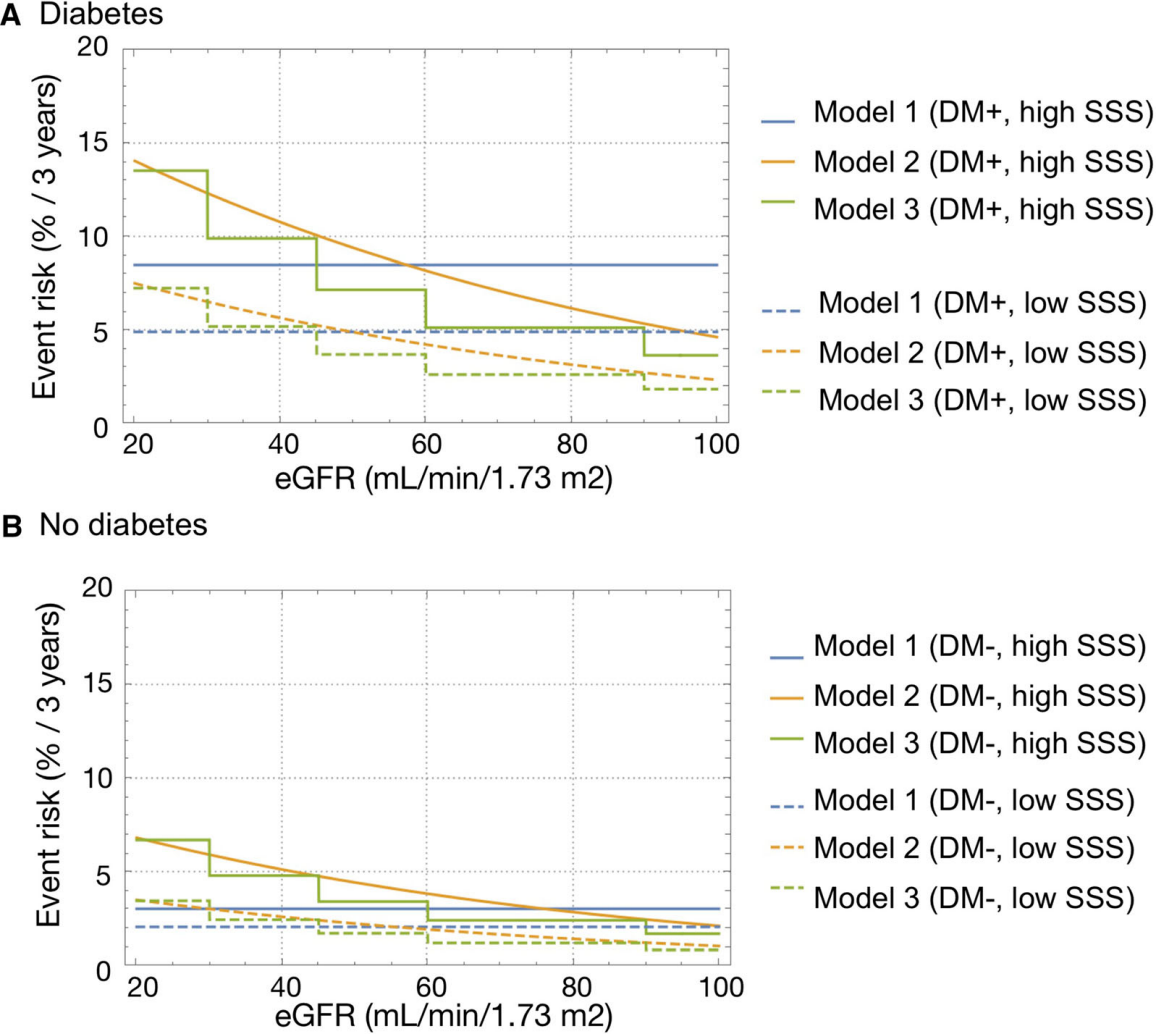
Comparison of three models depending on eGFR in a patient aged 60 years. With (+) and without (−) diabetes (DM), high/low summed stress score (SSS) and left ventricular ejection fraction 50%

Table 2 shows parameter estimates for the three J-ACCESS risk models.^10^,^11^ Figure 1 graphically shows an example of differences in estimated risk in a 60-year old patient with LVEF 50%. Event risk determined by Model 1 was not influenced by the eGFR values defined in the model, whereas calculated risk negatively depended on eGFR in Models 2 and 3. These graphs also indicated that the estimated risk was two-fold higher for patients with high (> 7) compared with low (≤ 7) SSS. Furthermore, complication with diabetes mellitus also increased risk two-fold.

### ROC Analysis for Predicting Cardiac Events

Areas under the ROC curve (AUC) were 0.66 (95% confidence of intervals [CI]: 0.58-0.73), 0.67 (0.59-0.73), and 0.66 (0.58-0.73) for Models 1, 2, and 3, respectively, indicating that the models similarly predicted low to high major cardiac event. Thresholds were determined from the highest value for sensitivity + specificity − 1 at 4.3, 5.2, and 4.5% risk for Models 1, 2, and 3, respectively.

### Survival Analysis and Event Rates in Three Risk Models

**Figure 2:**
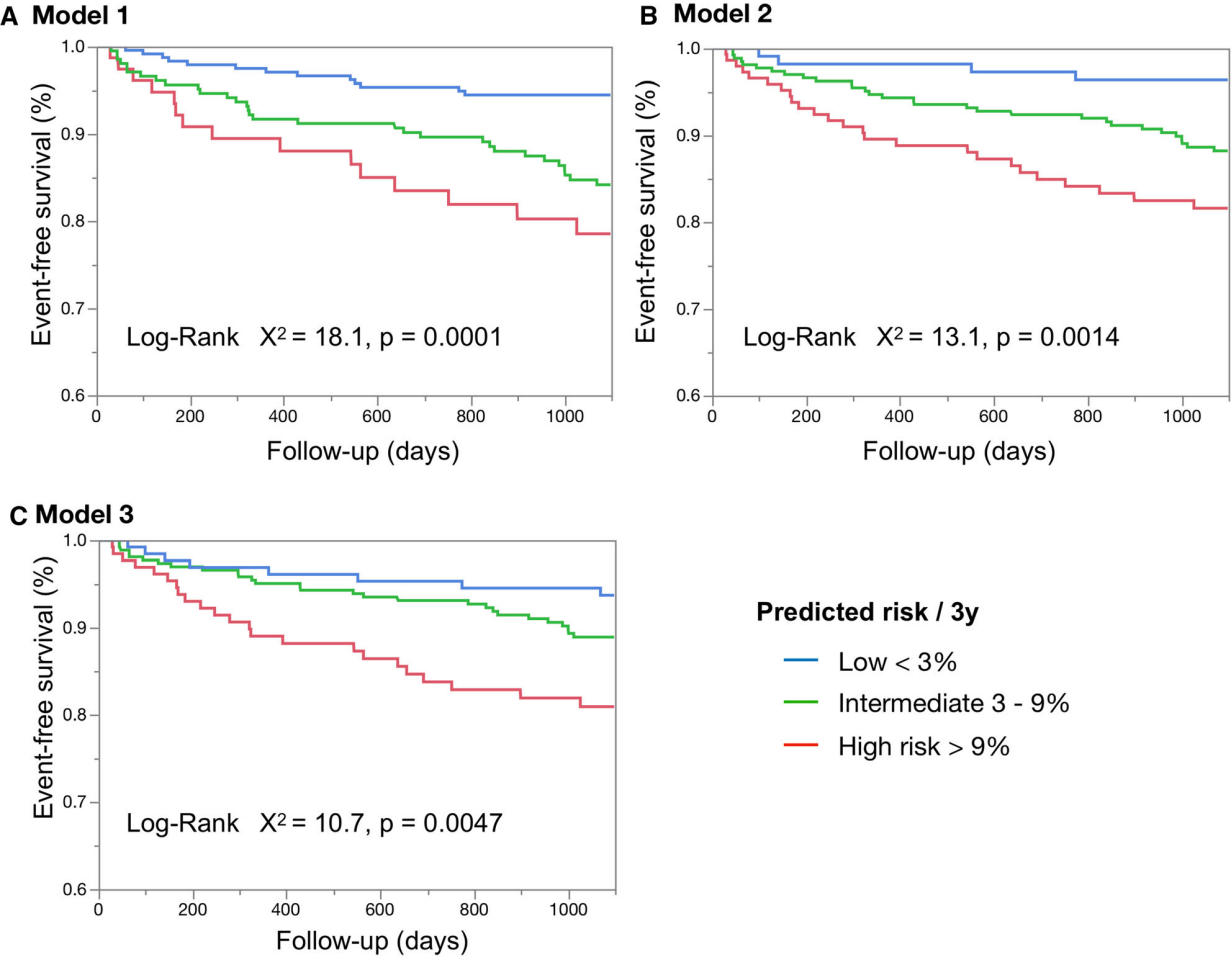
Survival analysis by three multivariable models. Model 1, without eGFR; Model 2, with continuous eGFR value and two SSS categories; Model 3, with categorical eGFR value and four SSS categories. Blue, green and red curves, patients at low (< 3%/3 y), intermediate (3%-9%/3 y) and high (> 9%) risk of developing cardiac events

Figure 2 shows that the groups with low (< 3%/3 y), intermediate (3%-9%/3y) and high (> 9%/3 y) risk for developing major events significantly differed among Models 1 (*P* = .0001), 2 (*P* = .0014), and 3 (*P* = .0047).

**Figure 3:**
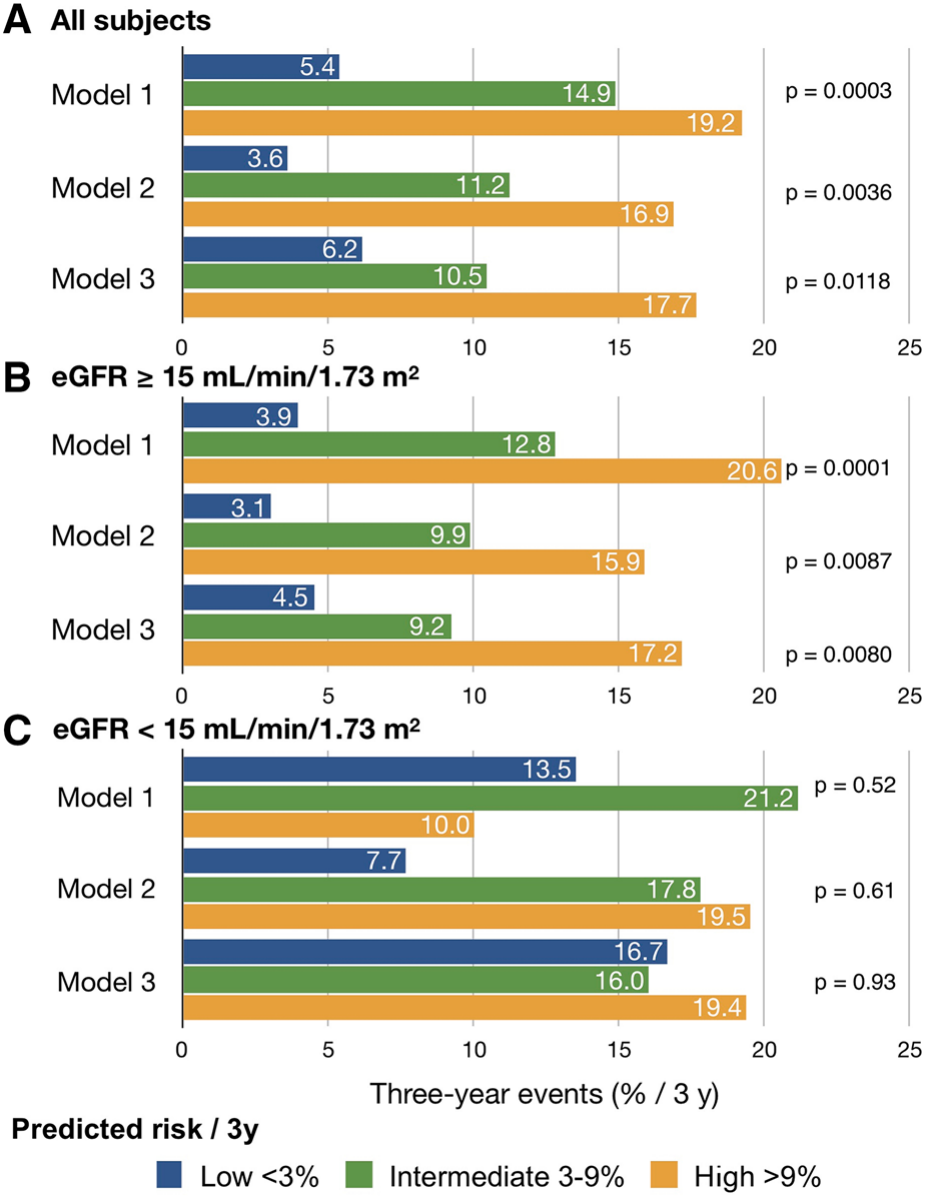
Three-year cardiac event outcome based on three models for patients at low (< 3%/3y), intermediate (3%-9%/3y) and high (> 9%/3y) risk. Panels (**A)**, (**B)** and (**C)** show all patients and those with eGFR ≥ 15 and < 15 mL/min/1.73 m^2^, respectively

Figure 3A shows that actual event rates significantly differed among the three risk groups. Figure 3B shows that when the patients were classified according to an eGFR threshold of 15 mL/min/1.73 m^2^, each risk group was clearly separated by the three models among those with eGFR ≥ 15 mL/min/1.73 m^2^. The actual event rate was the lowest among the low-risk (< 3%) patients in Model 2 (3.1%). In contrast, none of the three models could stratify patients with eGFR < 15 mL/min/1.73 m^2^ (*n* = 99) into three risk groups (Figure 3C).

### Frequency of High CRP in Risk Groups

**Figure 4:**
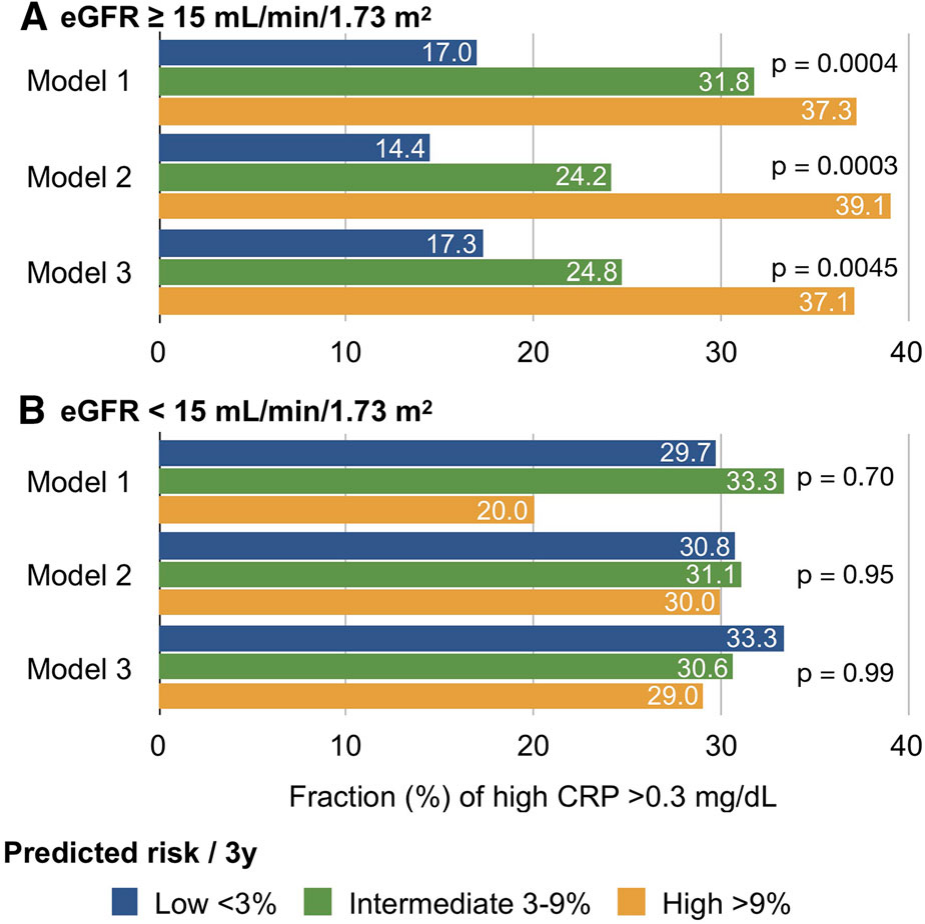
Frequency of high CRP (> 0.3 mg/mL) determined in three models among patients at low (< 3%/3y), intermediate (3%-9%/3y) and high (> 9%/3y risk of developing cardiac events. Panels A and B, patients with eGFR ≥ 15 and < 15 mL/min/1.73 m^2^, respectively

The CRP values ranged from 0 to 10.1 (median, 0.1; mean, 0.42 ± 0.98) mg/dL. Analyses of ROC curves showed that the optimal cutoff to obtain the highest sensitivity + specificity − 1 was 0.3 (AUC = 0.62, *P* = 0.0029). Figure 4 shows the frequency of high CRP (> 0.3 mg/mL) among patients at low, intermediate, and high risk using Models 1, 2, and 3. The frequency of high CRP was increased among patients with eGFR ≥ 15 mL/min/1.73 m^2^ depending on risk for all models (*P* = .0004, .0003, and .0045 for Models 1, 2, and 3, respectively). However, the frequency of elevated CRP was high in all groups of patients with eGFR < 15 mL/min/1.73 m^2^ regardless of model type.

## Discussion

The present study demonstrated the validity of the event risk models created by the J-ACCESS multi-center cohort study and presently applied in Japan.^10^,^11^ The estimation of major cardiovascular events has practical roles for deciding optimal treatment strategies for patients with suspected CAD.^1^,^3^,^17^ Actual event rates were higher than the risk predicted by the model created without eGFR but including eGFR improved risk assessment by the models. However, the incidence of cardiac events was higher regardless of predicted risk in patients with eGFR < 15 mL/min/1.73 m^2^, indicating a limitation of the risk model for such patients.

### J-ACCESS Risk Model

The J-ACCESS risk model validated herein was the first risk model created in Japan to estimate major cardiac events using quantitative gated stress-rest MPI.^18^ Based on multivariable logistic analysis, the model included the four variables of age, LVEF, SSS, and diabetes as predictor,^10^ and eGFR was subsequently added.^8^,^11^ Because SDS did not reach statistical significance in the multivariable analysis, it was not adopted in the model. Since respectively categorizing SSS and eGFR into four and five classes was considered clinically practical, risk calculation software that included these categorical variables was also created for clinical applications. The uniqueness of these risk models was that a multivariable logistics model provided major cardiac event risks (%) for 3 years. Therefore, agreement between the predicted risk and actual event rates was a concern and required validation. The model-derivation and validation populations differed insofar as the latter group had lower eGFR, SSS, and SRS. That is, our model was applied to patients with different demographics. The safety of pharmacological stress in patients with CKD was also confirmed.

### Validity of the Risk Models

The validation study showed that any of the three J-ACCESS risk models could stratify patients into being at low, intermediate, or high risk of developing major cardiac events. However, another important aspect of the model was the identical predicted and actual event rates (%). The risk of events predicted by Model 1 was underestimated compared with actual event rates (1%-3% vs 5.4% and 3%-9% vs 14.9%), whereas Model 2 including eGFR predicted 1%-3% and 3%-9% risk of events, compared with the actual rates of 3.1% and 9.9%, respectively. These outcomes were still slightly higher than the upper limit of the predicted values with eGFR. Although Models 2 and 3 tended to be similar, the risk of events predicted by Model 2 was 3.1%, which was lower than the 4.5% predicted by Model 3 for low-risk patients. Therefore, although the J-ACCESS risk model including eGFR improved risk estimation (%/3 years), the actual outcomes for patients with CKD remained underestimated to some degree.

### Estimated GFR as a Predictor of the Model

Predictive accuracy was quite different depending on eGFR > 15 (CKD stages 3a, 3b and 4 in the present study) or < 15 mL/min/1.73 m^2^ (stage 5), which was selected because this threshold is a significant predictor of survival in patients with CKD.^13^ Although the actual event rate was near the upper limit or slightly higher than the predicted rates in patients with ≥ 15 mL/min/1.73 m^2^, the rates were 3%-5% and 9%-13% for low- and intermediate-risk patients, respectively, whose predicted risk was apparently underestimated when eGFR was < 15 mL/min/1.73 m^2^. A comparison of Models 2 and 3 showed that Model 2 estimated lower risk for low-risk patients, and that the difference in outcome events among the risk groups tended to be larger than that of Model 3. This could be explained by differences between the numerical and categorical applications of variables; eGFR < 30 mL/min/1.73 m^2^ was categorized into one category in Model 3, but continuous in Model 2. These model structures might have caused the larger difference in calculated risk at lower eGFR.

### High-Risk Patients with Low eGFR

The underestimated risk among patients with CKD stage 5 (eGFR < 15 mL/min/1.73 m^2^) is an important limitation of the present risk model. Since few patients (3%) had stage 5 CKD (end-stage) in the model-derivation group, the predictive accuracy of the model might be less accurate with respect to this validation group. Although the present study could not determine the pathophysiological reasons for this phenomenon, high CRP or an inflammatory reaction might have been associated, which has also been noted in previous studies. Potentially critical elements in the initiation, progression, and rupture of plaque have become evident, and anti-inflammatory and antioxidant therapies have been sought.^19^ In fact, the distribution of event outcomes and positive CRP were related in the present study (Figures 3, 4), as CRP was elevated at the initiation of dialysis treatment among some patients with CKD and it is a predictor of cardiac events.^20^,^21^ Analysis of relationships among metabolic syndrome, high-sensitivity CRP, and CKD revealed that CRP is a powerful risk factor for arterial stiffness, cardiovascular events, and mortality.^22^ In the present validation population, the high proportion of patients with severe HF requiring hospitalization might be a result of these complex backgrounds. Moreover, when renal events were analyzed during the 3-year follow-up, 44 of 99 patients required either hemodialysis or peritoneal dialysis. Progression to renal insufficiency could exacerbate cardiac function, leading to chronic HF. However, the long-term effects of hemodialysis are beyond the scope of the present study and will require a follow-up investigation. Another factor might be associated with sympathetic derangement in patients with severe CKD. Cardiac ^123^I-metaiodobenzylguanidine uptake was low among patients with low eGFR, and this contributed to the high cardiac mortality risk among patients with chronic HF.^23^

## Limitations

The present study included patients with CKD stages 3 to 5, whereas the model-derivation population included stages 1 to 5. Although stages 1 and 2 were not included in the validation population, including low-risk patients with an annual event rate of < 1% (21% and 25% of patients determined by Models 2 and 3, respectively), a wide range of risk was evaluated. A recent validation of J-ACCESS Model 2 also found that an event risk of 10% separated the prognosis of patients with a mean eGFR of 67.4 ± 24.3 mL/min/1.73 m^2^.^24^ Therefore, although the present study focused on patients with CKD, the J-ACCESS model could apply even to patients with normal eGFR. In addition, further validation studies are needed to clarify the relevance of including additional variables such as CRP to enhance predictive accuracy.

## New Knowledge Gained

Three J-ACCESS models were applied to assess risk of major cardiac events among CKD patients in the J-ACCESS-3 study. Although all risk models for major cardiac events could stratify outcomes of patients with CKD, the model that included eGFR was more appropriate for these patients. Risk stratification was effective for patients with stages 3a, 3b, and 4 CKD (eGFR ≥ 15 mL/min/1.73 m^2^), whereas patients with stage 5 CKD (eGFR < 15 mL/min/1.73 m^2^) are potentially at high risk across all estimated risk values.

## Electronic supplementary material

Below is the link to the electronic supplementary material.

Supplementary material 1 (PPTX 839 kb)

## Abbreviations

CAD: Coronary artery disease
CKD: Chronic kidney disease
eGFR: Estimated glomerular filtration rate
HF: Heart failure
LVEF: Left ventricular ejection fraction
MI: Myocardial infarction
SSS: Summed stress score
