# Supplementary material for: Risk stratification based on J-ACCESS risk models with myocardial perfusion imaging: Risk versus outcomes of patients with chronic kidney disease
Source: J Nucl Cardiol. 2018 Jun 12;27(1):41–50. doi: 10.1007/s12350-018-1330-8 (PMC7031191; doi:10.1007/s12350-018-1330-8)
Supplement: Supplementary file 1 — Supplementary material 1 (PPTX 839 kb) [file 12350_2018_1330_MOESM1_ESM.pptx]

## Slide 1
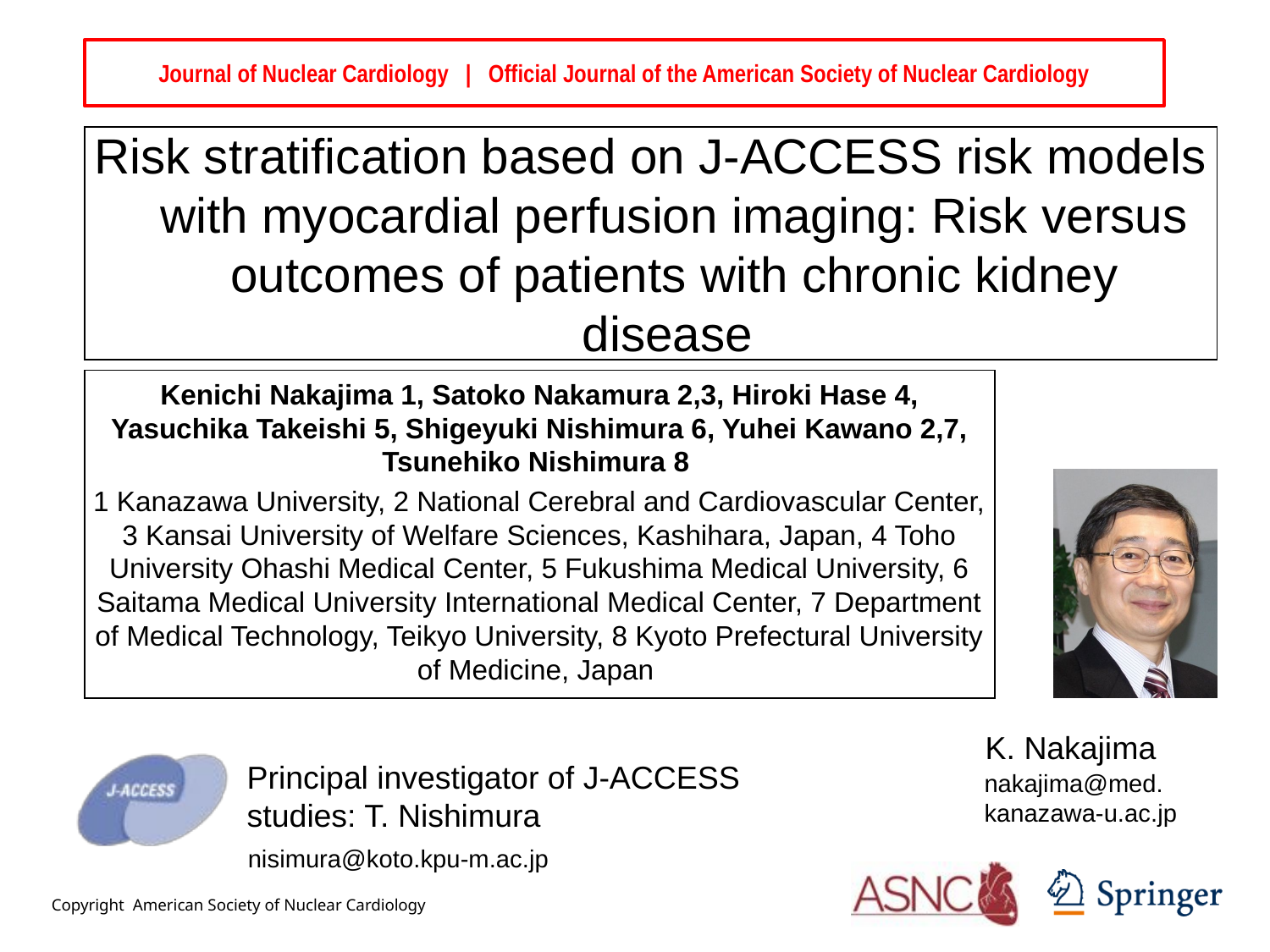

Journal of Nuclear Cardiology | Official Journal of the American Society of Nuclear Cardiology
# Risk stratification based on J-ACCESS risk models with myocardial perfusion imaging: Risk versus outcomes of patients with chronic kidney disease
Kenichi Nakajima 1, Satoko Nakamura 2,3, Hiroki Hase 4, Yasuchika Takeishi 5, Shigeyuki Nishimura 6, Yuhei Kawano 2,7, Tsunehiko Nishimura 8
1 Kanazawa University, 2 National Cerebral and Cardiovascular Center, 3 Kansai University of Welfare Sciences, Kashihara, Japan, 4 Toho University Ohashi Medical Center, 5 Fukushima Medical University, 6 Saitama Medical University International Medical Center, 7 Department of Medical Technology, Teikyo University, 8 Kyoto Prefectural University of Medicine, Japan
K. Nakajima
Principal investigator of J-ACCESS studies: T. Nishimura
nakajima@med. kanazawa-u.ac.jp
nisimura@koto.kpu-m.ac.jp
Copyright American Society of Nuclear Cardiology

## Slide 2
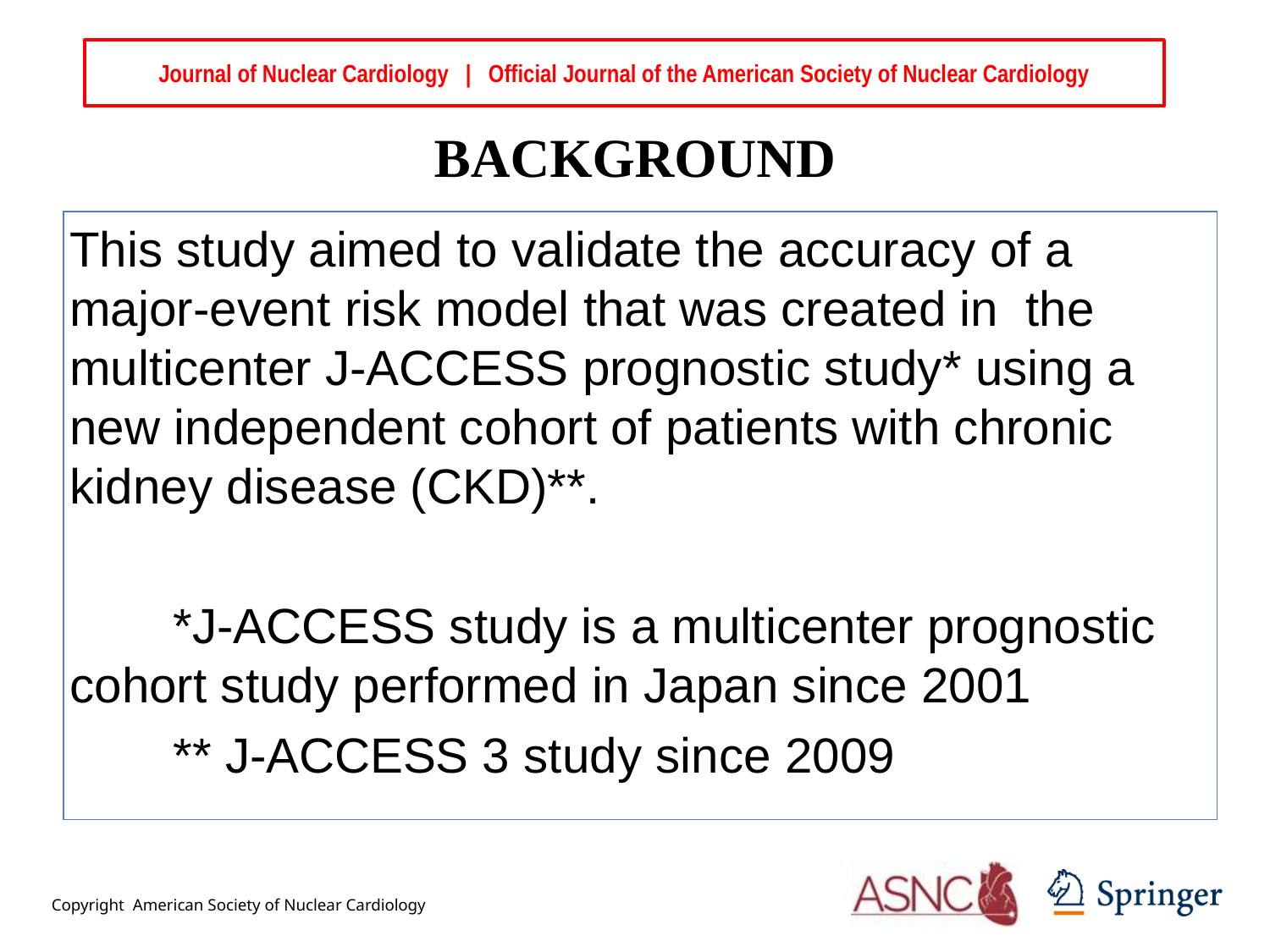

Journal of Nuclear Cardiology | Official Journal of the American Society of Nuclear Cardiology
# BACKGROUND
This study aimed to validate the accuracy of a major-event risk model that was created in the multicenter J-ACCESS prognostic study* using a new independent cohort of patients with chronic kidney disease (CKD)**.
*J-ACCESS study is a multicenter prognostic cohort study performed in Japan since 2001
** J-ACCESS 3 study since 2009
Copyright American Society of Nuclear Cardiology

## Slide 3
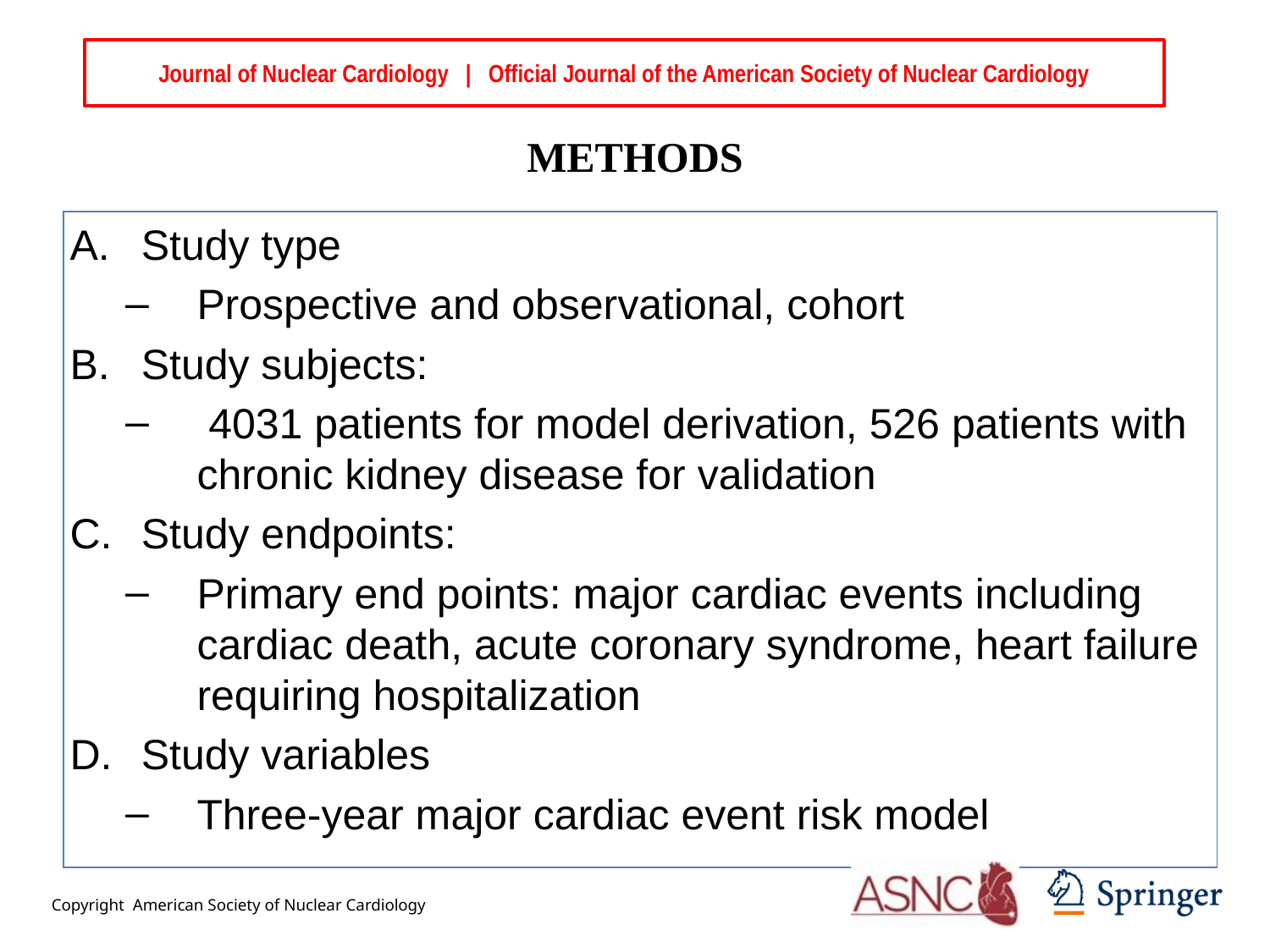

Journal of Nuclear Cardiology | Official Journal of the American Society of Nuclear Cardiology
# METHODS
Study type
Prospective and observational, cohort
Study subjects:
 4031 patients for model derivation, 526 patients with chronic kidney disease for validation
Study endpoints:
Primary end points: major cardiac events including cardiac death, acute coronary syndrome, heart failure requiring hospitalization
Study variables
Three-year major cardiac event risk model
Copyright American Society of Nuclear Cardiology

## Slide 4
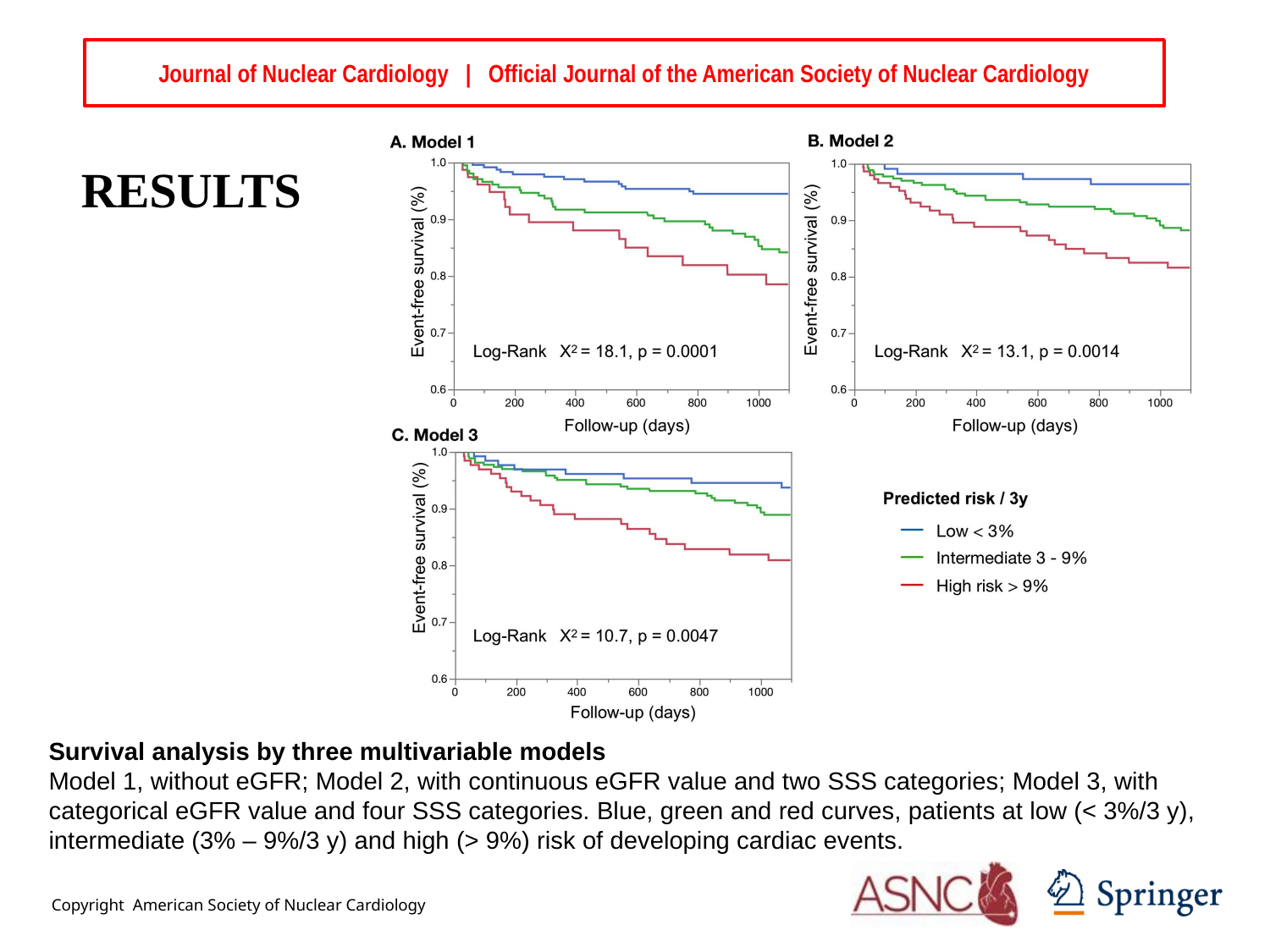

Journal of Nuclear Cardiology | Official Journal of the American Society of Nuclear Cardiology
# RESULTS
Survival analysis by three multivariable models
Model 1, without eGFR; Model 2, with continuous eGFR value and two SSS categories; Model 3, with categorical eGFR value and four SSS categories. Blue, green and red curves, patients at low (< 3%/3 y), intermediate (3% – 9%/3 y) and high (> 9%) risk of developing cardiac events.
Copyright American Society of Nuclear Cardiology

## Slide 5
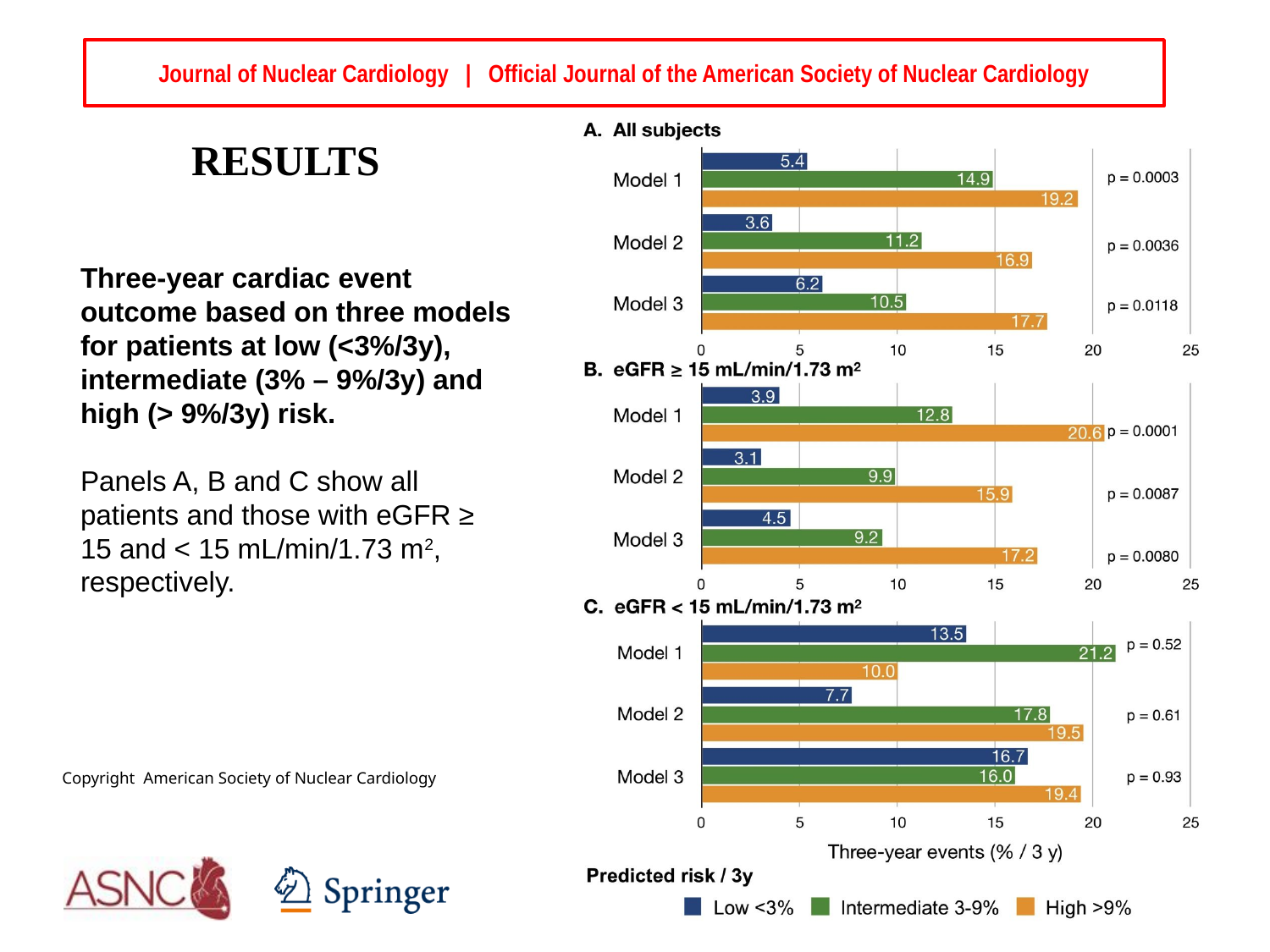

Journal of Nuclear Cardiology | Official Journal of the American Society of Nuclear Cardiology
# RESULTS
Three-year cardiac event outcome based on three models for patients at low (<3%/3y), intermediate (3% – 9%/3y) and high (> 9%/3y) risk.
Panels A, B and C show all patients and those with eGFR ≥ 15 and < 15 mL/min/1.73 m2, respectively.
Copyright American Society of Nuclear Cardiology

## Slide 6
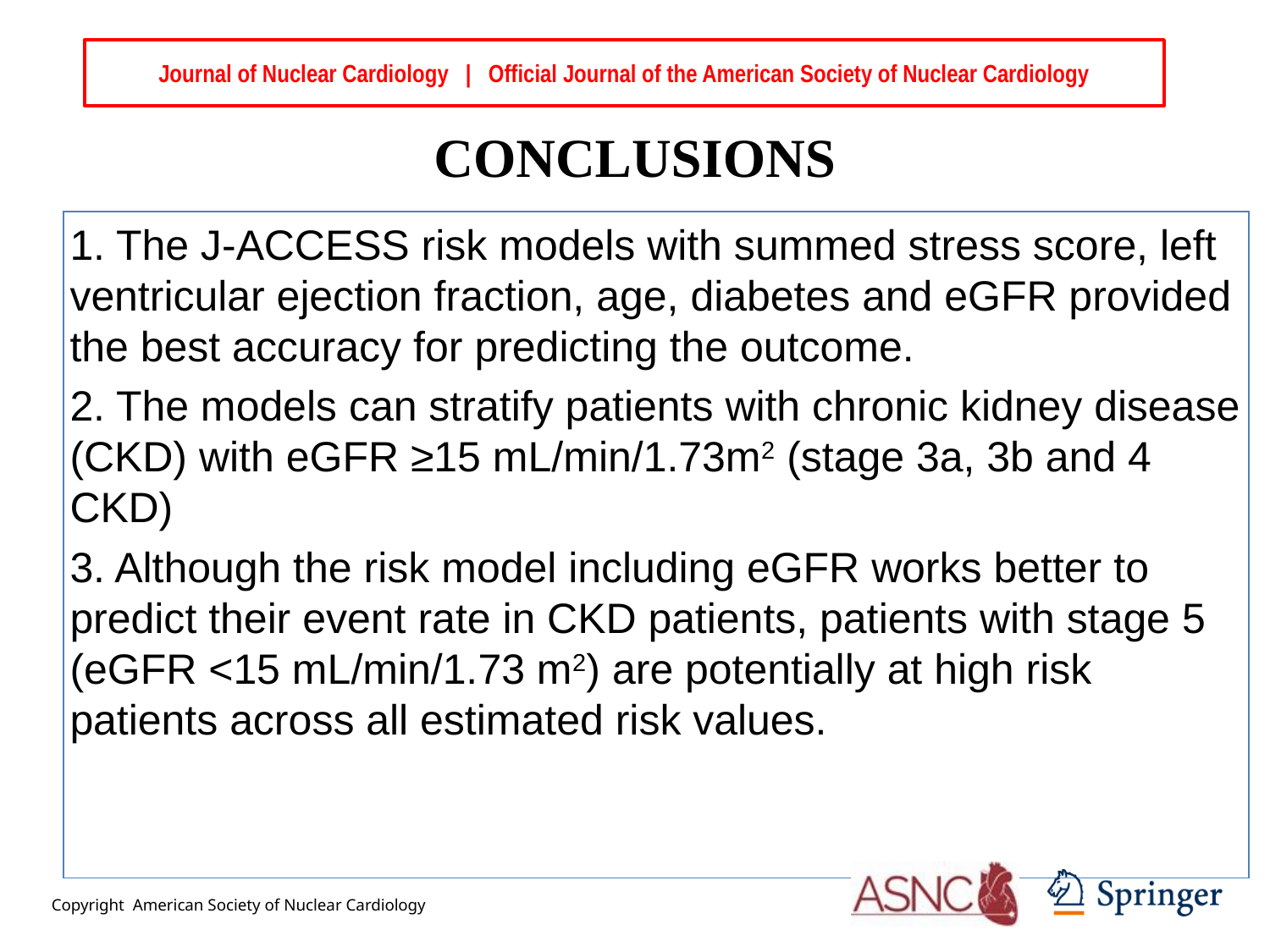

Journal of Nuclear Cardiology | Official Journal of the American Society of Nuclear Cardiology
# CONCLUSIONS
1. The J-ACCESS risk models with summed stress score, left ventricular ejection fraction, age, diabetes and eGFR provided the best accuracy for predicting the outcome.
2. The models can stratify patients with chronic kidney disease (CKD) with eGFR ≥15 mL/min/1.73m2 (stage 3a, 3b and 4 CKD)
3. Although the risk model including eGFR works better to predict their event rate in CKD patients, patients with stage 5 (eGFR <15 mL/min/1.73 m2) are potentially at high risk patients across all estimated risk values.
Copyright American Society of Nuclear Cardiology
